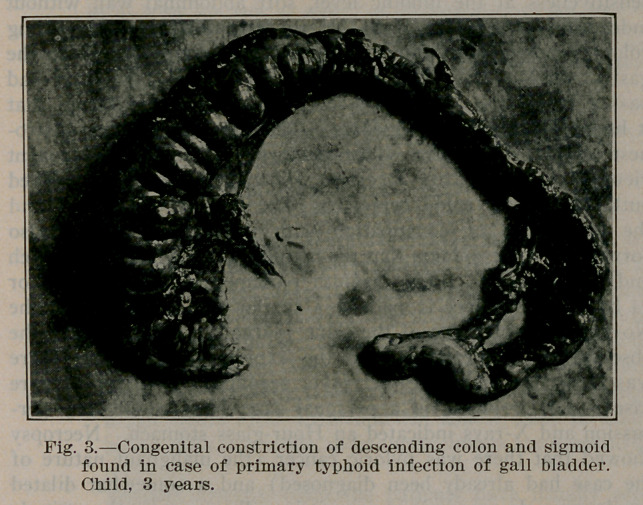# A Case of Cholecystitis

**Published:** 1913-05

**Authors:** 


					﻿A Case of Cholecystitis due to infection of the gall bladder
by the typhoid bacillus in a child three years old. Case report
by H. Lowenburg, A. M., M. D., Philadelphia, Arch, of Paed.,
March, 1913. (Cut kindly loaned by Journal).
				

## Figures and Tables

**Fig. 3. f1:**